# Accuracy for diagnosis of periapical cystic lesions

**DOI:** 10.1038/s41598-020-71029-3

**Published:** 2020-08-25

**Authors:** Igor Tsesis, Gal Krepel, Tal Koren, Eyal Rosen, Anda Kfir

**Affiliations:** Department of Endodontology, Maurice and Gabriela Goldschleger School of Dental Medicine, Tel Aviv University, Tel Aviv, Israel

**Keywords:** Diseases, Medical research, Pathogenesis

## Abstract

Clinical differentiation between cystic lesions of endodontic and non-endodontic origin is of importance because correct diagnosis may affect treatment decision making. Most radicular cysts are treated with conservative approaches and, therefore, are not surgically removed. The objective of this study was to determine the accuracy of clinical diagnosis of periapical lesions as compared to the histological findings, and to evaluate various associated factors. All biopsy specimens submitted for histological evaluation from 2002 to 2009 were assessed. Only cases of periapical lesions with complete patient data and clinical diagnosis were included. Sensitivity, specificity and accuracy of the clinical diagnosis were calculated and various patient-related factors were evaluated. Of the 4,908 cases, 183 met inclusion criteria. Histologically, there were 171 lesions of radicular cysts and 12 cases of non-endodontic cysts, including OKC and Incisive Canal Cyst. The diagnostic accuracy for clinical diagnosis for radicular cysts was 91.84% and 91.84% for non-endodontic cysts. There was a high accuracy of clinical differentiation between cystic lesions of endodontic and non-endodontic origin. However, some non-endodontic lesions may be incorrectly diagnosed clinically as lesions of endodontic origin. Histological evaluation may be necessary for the correct diagnosis. Further clinical studies are needed to evaluate clinical examination and histological diagnosis of periapical lesions.

## Introduction

Periapical lesions are most commonly of endodontic origin and related to pulp infection^1,2^. Bacteria and their by-products can exit the root canal system through the apical foramen and cause an inflammatory response in the periapical tissues^3–5^ and resorption of the alveolar bone surrounding the root^6^. Most lesions of endodontic origin can be classified as periapical granuloma or radicular cyst^7–12^. The reported prevalence of radicular cysts within periapical lesions varies between 6 and 55% and of periapical granulomas ranges between 46 and 84%^7–13^.

The most of the cysts in the jaws develop from odontogenic epithelium and classified as inflammatory and developmental^14–16^.

The origin of the inflammatory cysts are the epithelial rests of Malassez. The bacterial byproducts from the contaminated necrotic pulp may stimulate the proliferation of these epithelial rests and lead to the formation of a radicular cyst^3,4,10,17^. Radicular cysts are the most common cysts found in the jaws. Their epithelial lining may demonstrate varying degrees of inflammation; additionally, cholesterol crystals and fibrosis may be found in the cystic cavity^10,13,18^.

Among the cysts of the developmental origin are Dentigerous cysts and Keratocystic Odontogenic Tumor (KCOT). Dentigerous cysts are commonly found in children from 2 to 14 years. KCOT are often found in the posterior mandible. They are considered aggressive and have a higher recurrence rate relatively to the other odontogenic cysts^19^, and require surgical treatment.

Determination of the cysts’ nature is of major importance. Most teeth undergoing periapical surgery are diagnosed as previously treated, therefore pulp sensitivity testing, though frequently used to distinguish between apical lesions of endodontic and non-endodontic origin, becomes redundant. The difference in prognosis and treatment of periapical lesions not responding to endodontic treatment, has led to controversy regarding the possibility of submitting periapical specimens for histopathologic examination^1^. There is also controversy regarding the ability of radiographical examination to identify lesions accurately^20,21^.

The aim of this study was to determine the accuracy of clinical diagnosis of periapical cystic lesions in comparison with histologic findings, and to evaluate various associated factors.

## Materials and methods

All biopsy specimens submitted to the oral pathology department between the years 2002 and 2009 were reviewed. The study was approved by Tel Aviv University the ethics committee (IRB reference number 1810.10). Informed consent was obtained from all subjects. All methods were carried out in accordance with relevant guidelines and regulations. Only cases of biopsy of periapical area adjacent to endodontically treated teeth were included. The collected specimens included biopsies following teeth extraction, apical surgery and cyst enucleation. Only cases with detailed clinical information including age, gender, clinical diagnosis, location of lesion, size of lesion were included. Cases without a clinical diagnosis or radiographic evaluation and cases that were not diagnosed histologically as radicular cyst (RC) or non-endodontic cysts (NEC) were excluded from the study. The biopsy tissue samples were fixed in 10% buffered formalin and embedded in paraffin. For each case, random 5-µm sections were cut and stained with hematoxylin and eosin, periodic acid—Schiff, and Gram stain. 3 random sections were analyzed per sample.

The radiographic cystic lesion size was measured and the mean height by width was obtained in millimeters. The cystic lesions were classified according to size as following:

- small (< 10 mm)
- large (> 10 mm)

We classified small lesion from large lesion based on the threshold used in previous researches^22,23^.

The results were evaluated statistically as following.

The association between the clinical diagnoses and histological diagnoses, gender, lesion size and lesion location were analyzed using chi-squared test analysis.

Association between the age of the patient and clinical and histological diagnoses was analyzed using t tests for descriptive numeric data.

Sensitivity, specificity and accuracy of the clinical diagnosis were calculated. Sensitivity was defined as proportion of people who test positive for the disease among those who have the disease. Specificity was defined as the proportion of healthy patients known not to have the disease, who will test negative for it.

In the present study the sensitivity was defined as radicular cyst and the specificity was defined as non-endodontic cysts.

Accuracy was defined as the proportion of both true positives and true negatives cases among the total number of cases examined.

The p-value was set at 0.05.

## Results

**Figure 1:**
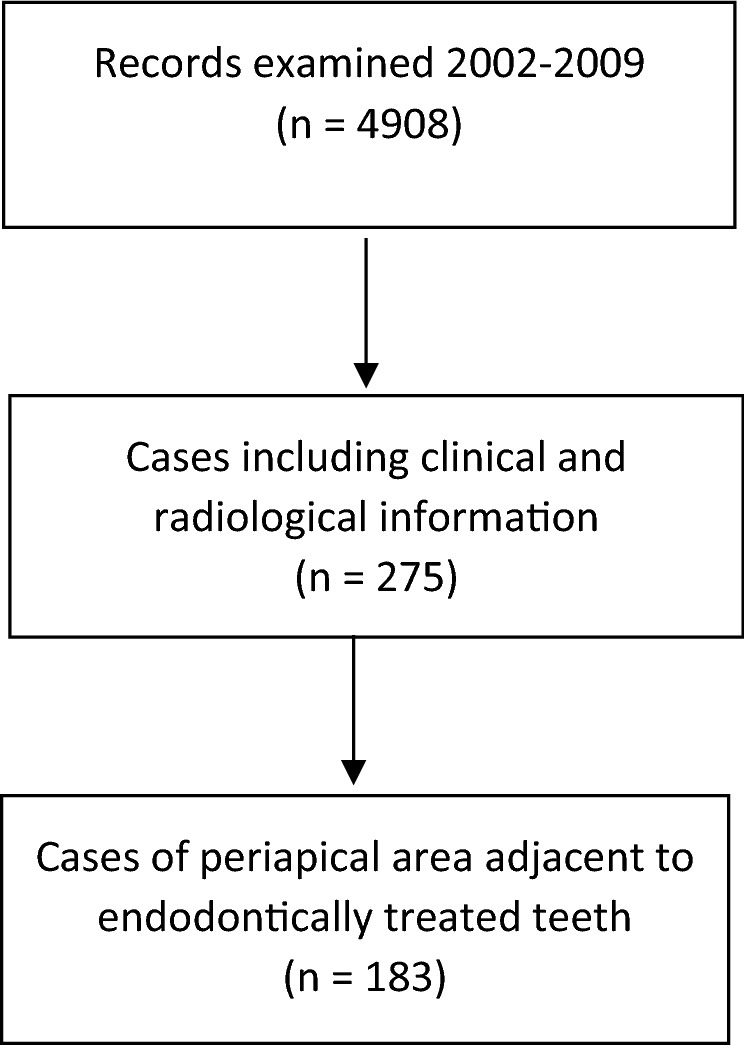
Flow diagram for cases included.

Of the 4,908 cases examined, 183 cases met the criteria for inclusion (Fig. 1).

Mean patient age was 40.16 years, with a 54.6% male predilection. No association was found between age and gender and between clinical and histological diagnoses. Of the 183 cases, 89 (48.9%) were located in the mandible and 94 (51.1%) were located in the maxilla.

Histologically; 171 cases (93.4%) were radicular cyst, and 12 cases (6.6%) were NEC.

NEC consisted of 11 cases of KCOTs and one case of incisive canal cyst.

There was significant association between clinical and histological diagnoses. Cases of RC were clinically diagnosed correctly in 89.1% while cases of NEC, were clinically diagnosed correctly in 41.7% (p < 0.05) (Table 1).

**Table 1:** Relationship between clinical and histological diagnoses of RC and NEC.

	Clinical diagnosis		
	RC	NEC	Total
**Histological diagnoses**			
RC	164 95.9%	7 4.1%	171
NEC	6 54.5%	5 45.5%	11

*RC* radicular cysts, *NEC* non-endodontic cysts.

**Figure 2:**
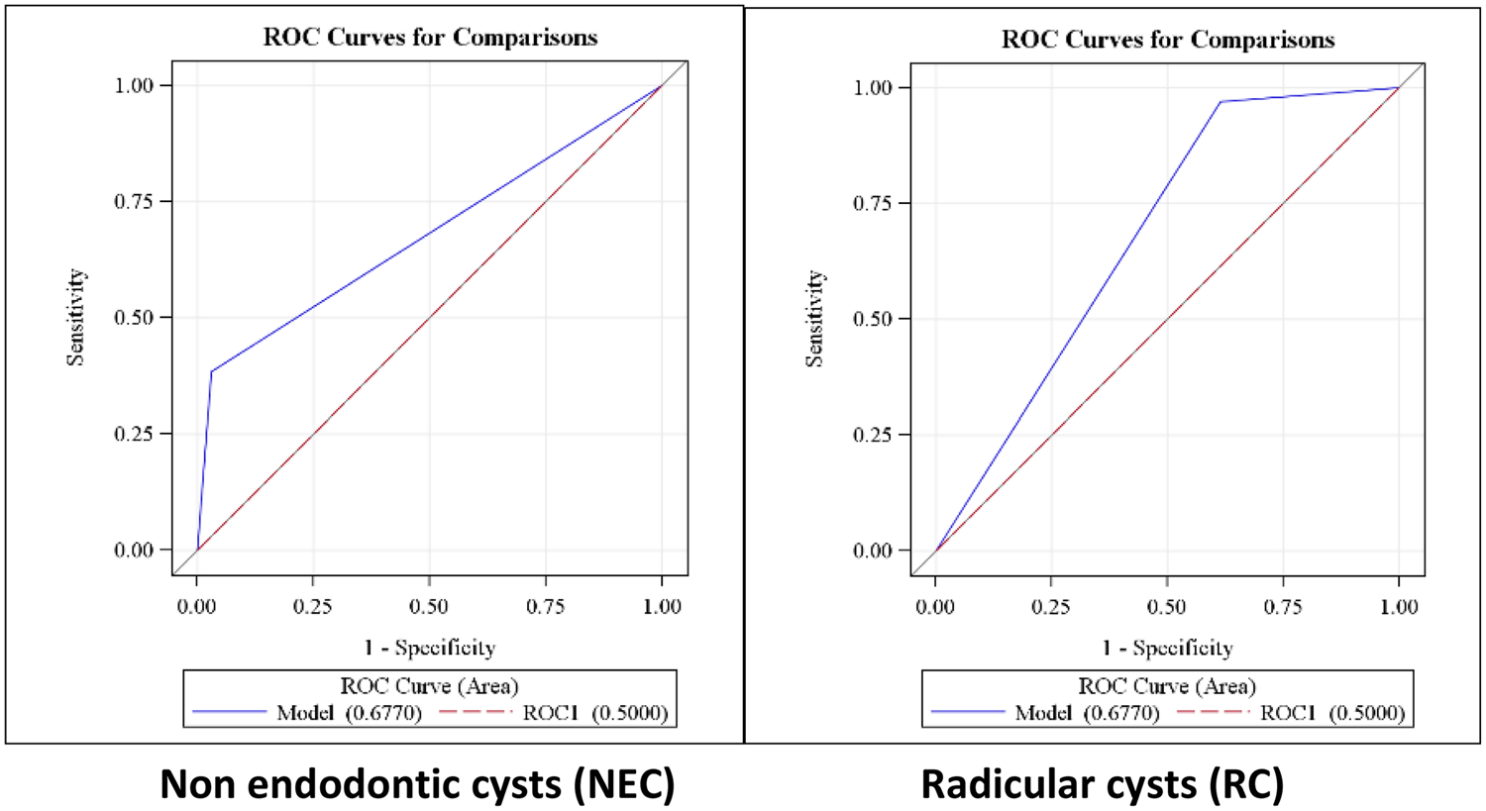
ROC charts for RC and NEC.

Of the 183 cases, 159 (86.5%) were classified as large, 24 (13.5%) were classified as small. For large lesions there was a significantly higher prevalence of both radicular cysts and NEC (p < 0.05),

Sensitivity, specificity and accuracy for clinical diagnosis are presented in Table 2 and ROC charts are presented in Fig. 2.

**Table 2:** Sensitivity, specificity, PPV, NPV and accuracy for clinical diagnosis.

Accuracy (%)	NPV (%)	PPV (%)	Specificity (%)	Sensitivity (%)
92.8	41.6	96.5	45.5	95.9

*PPV* positive predictive value, *NPV* negative predictive value.

## Discussion

In the present study, the association between the clinical and histological diagnoses was evaluated in 184 cases.

However, the biopsy specimens used in the present study were curetted and thus fragmented in several cases, making histological differentiation inaccurate. Periapical granulomas were diagnosed when there was no clear epithelial-lined lumen, and were not included in the present study. Moreover, it was not possible to evaluate with certainty the communication between the biopsy specimens and the root canal, due to the fact that the specimens did not contain root tips.

Kontogiannis et al.^24^ found that NEC constituted 3.42% of the total cases of periapical lesions. These findings correspond with the findings in the present study, in which 6.6% of the cases were NEC.

Jones et al.^13^, evaluated the demographics of odontogenic cysts diagnosed in a UK population over a 30-year period, and found that out of 7,121 specimens that were diagnosed as odontogenic cysts, 3,724 (52.3%) were radicular cysts. These findings correspond with the present study, in which 58.3% (7 cases out of 12) of NEC were histologically diagnosed as radicular cysts.

The present study indicates that the diagnostic accuracy for clinical diagnosis for radicular cysts was 91.84%.

Other studies of periapical biopsy specimens have suggested that in 0.7–5% of the cases there were differences between clinical and histological diagnoses^1,3,4,25^. However, these studies have not calculated the accuracy or the sensitivity of the diagnosis.

The present study refers to the clinical diagnostic accuracy for radicular cysts as opposed to NEC whereas.

Stockdale et al.^25^ calculated the difference in accuracy between radicular cysts and periapical granuloma. They found that the clinical diagnostic accuracy for radicular cysts was 41% and that the clinical diagnostic accuracy for periapical granuloma was 81.4%, which they consider to be a relatively high. We can conclude that the current clinical and radiological processes for distinguishing between periapical granuloma and radicular cysts is not accurate enough.

The present study indicates that for large lesions there was a significantly higher prevalence of both radicular cysts (83%) and NEC. Mortensen et al.^22^ indicated that lesions larger than 15–20 mm, can be safely classified as cysts. However, various studies have indicated that basing diagnoses on radiographic analysis is not enough. Matsuda et al.^26^ concluded that only through clinical and radiographic examination it is not possible to confirm the diagnosis of lesions.

Clinical differentiation between cystic lesions of endodontic and non-endodontic origin is of importance because correct diagnosis may affect treatment decision making. Most radicular cysts are treated with conservative approaches (endodontic treatment) and, therefore, are not surgically removed^19^. Furthermore, radicular cysts, are related to preventable causes (such as infections) and actions to promote oral health may help to reduce the prevalence of these lesions^27^.

This paper calculated the accuracy, sensitivity and specificity for differentiating between cystic lesions of endodontic and non-endodontic origin. We can conclude from our findings that while the current accuracy of clinical and radiological processes for distinguishing between the various periapical pathoses is high, the gold standard for diagnosis and identification of periapical lesions is histological examination.

## Conclusions

There was a high accuracy of clinical differentiation between cystic lesions of endodontic and non-endodontic origin. However, some non-endodontic lesions may be incorrectly diagnosed clinically as lesions of endodontic origin. Histological evaluation may be necessary for the correct diagnosis.

Further clinical studies are needed to evaluate clinical examination and histological diagnosis of periapical lesions.
